# Impact of *Pleurotus ostreatus* β-Glucans on Oxidative Stability of Active Compounds Encapsulated in Powders during Storage and In Vitro Digestion

**DOI:** 10.3390/antiox9121219

**Published:** 2020-12-03

**Authors:** Francesca Gallotti, Anaïs Lavoisier, Christelle Turchiuli, Vera Lavelli

**Affiliations:** 1 DeFENS, University of Milan, 20133 Milan, Italy; vera.lavelli@unimi.it; 2 UMR SayFood, Université Paris-Saclay, INRAE, AgroParisTech, 91300 Massy, France; anais.lavoisier@inrae.fr (A.L.); christelle.turchiuli@agroparistech.fr (C.T.); 3 Department Chimie, Université Paris-Saclay, IUT d’Orsay, 91400 Orsay, France

**Keywords:** oxidative stability, in vitro digestion, β-glucans, antioxidant activity, sustainability

## Abstract

Polyunsaturated fatty acids and α-tocopherol were encapsulated in powders by spray drying using maltodextrins DE 12 as wall material and different emulsifiers (Tween^®^20, acacia gum or β-glucans-rich extracts from *Pleurotus ostreatus*). The aim was to study the effects of the surfactants on: (a) the oil droplet size distribution and α-tocopherol stability during in vitro digestion, and (b) the oxidative stability during 15 days of accelerated storage. Acacia gum sample had the most stable particle size distribution up to the gastric phase, but showed a significant α-tocopherol degradation prior to the intestinal stage. On the contrary, β-glucan-samples displayed a bimodal distribution in the oral and gastric phases but retained α-tocopherol up to the beginning of the intestinal stage. At the end of intestinal stage, no α-tocopherol was found in the samples. The storage study showed that β-glucans improved the oxidative stability of the powders, which displayed 82% α-tocopherol retention after 5 days under accelerated conditions (60 °C), corresponding to 310 days at 20 °C, while acacia gum and Tween^®^ 20 did not delay α-tocopherol degradation. Results highlight the potential antioxidant activity of β-glucans used as emulsifying agents during in vitro digestion and accelerated aging conditions.

## 1. Introduction

The importance of oils and their lipophilic compounds lies in their multifunctional role. They have an impact on the organoleptic properties of food products, varying their taste, appearance, flavour, texture and shelf life, and they are also necessary for the maintenance of the function and structure of the human body and for the preservation of well-being [1]. In particular, lipophilic compounds rich in large unsaturated hydrocarbon chains, named polyunsaturated fatty acids (PUFAs), are responsible for exercising various biological action, such as preserving cell-membrane fluidity, decreasing secretion of pro-inflammatory cytokines by monocytes/macrophages, reducing vulnerability to ventricular rhythm disorders of the heart, inhibiting inflammatory processes, preventing blood platelet aggregation, reducing triglyceride synthesis in the liver and improving functions of vascular endothelial cells [2]. Sunflower oil (SO) contains a considerably high amount (60%) of PUFAs, which are more prone to lipid oxidation than saturated ones, mainly due to their low activation energy for fatty acid radicals formation [3]; oxidation of PUFAs is damaging to cell function and leads to instabilities in membrane function and structure. Therefore, it is important to consume foods rich in antioxidants, since they are potentially able to quench or neutralize excess radicals. Vitamin E is the main lipid-soluble antioxidant in the cell antioxidant defence system and is entirely obtained from the diet. There are eight possible isomers of vitamin E, but α-tocopherol (5,7,8-trimethyltocol) (α-toc) is the most biologically important antioxidant in vivo [4]. SO contains up to 59 mg of α-toc per 100 g of oil, more than the other commonly consumed vegetable oils, i.e., palm, soybean and canola oil [5]. The major biological role of vitamin E is to protect PUFAs and other components of cell membranes from oxidation by being preferentially oxidized by free radicals. However, the amount of α-toc in biological membranes is approximately one part per 1000 lipid molecules, and the replenishment of α-toc is primarily achieved through dietary food components. Consequently, the protection of α-toc against factors promoting its degradation is critical to maintain efficacious concentrations of this bioactive compound in foods [6].

Encapsulation in powder improves the stability of the functional properties of lipophilic compounds by adding a physical barrier. The first step to encapsulate lipophilic compounds in powder consists in the preparation of a stable oil-in-water (O/W) liquid emulsion. Therefore, the choice of wall material and emulsifying agent is of great importance in order to maximize the incorporation and retention of the functional compounds [7]. Maltodextrins (MD), for example, are relatively low-cost polysaccharides with high solubility in water, low viscosity at high solids concentrations (e.g., up to 40–60% *w/w* dry matter), neutral taste and aroma and that act as an effective wall material [7,8]. However, since MD have no interfacial properties, they must be used in association with surfactants, such as polyoxyethylene (20) sorbitan monolaurate (Tween^®^20), or emulsifiers, such as acacia gum (AG), to produce physically stable emulsions. Several researchers have studied the use of mixture of these coatings to encapsulate lipophilic bioactive compounds [1,8,9,10]. In addition to the physical protection provided by encapsulation, the usage of compounds with antioxidant properties in the wall material, such as β-glucans, can make a valuable contribution for the protection and preservation of food. Antioxidant activity of β-glucans derived from microorganisms such as *Paenibacillus polymyxa* and mushrooms such as *P. ostreatus* have been studied [11,12]. In a preliminary study, β-glucans-rich extracts from *P. ostreatus* were successfully used both for the preparation of stable O/W liquid emulsions and for the protection, during spray drying, of SO encapsulated in them. The efficiency regarding oil protection was evaluated by measuring both the amount of conjugated dienes (CD) (as evaluation of the primary oxidation compounds) and α-toc after spray drying, with positive results [7].

Oxidative degradation of food lipids also occurs during digestion because of the pro-oxidant conditions of the gastrointestinal tract (GIT) (oxygen incorporated during mastication, low pH of gastric juice, and presence of reactive species like metallic ions). Moreover, the products of the enzymatic hydrolysis of food lipids are prone to oxidative degradation [13]. Consequently, the bioavailability of lipid-based components of nutritional interest depends on multiple factors such as the lipid content of the food, the unsaturation degree, and the initial oxidative status of the lipids, the presence of phenolic compounds or proteins in the food bolus, and processing conditions before ingestion, among others [13]. Oxidative loss of tocopherols during in vitro digestion has also been reported by Kenmogne-Domguia et al. [14], which could be prevented by the addition of protective compounds with antioxidant properties, like β-glucans rich extracts from *P. ostreatus.*

Besides the protection from degradation during digestion, it is crucial that a bioactive compound has a high bioavailability after ingestion, so that it can effectively deliver its positive biological effects [15]. Bioavailability of lipophilic vitamins, such as vitamin E, encapsulated in O/W emulsions is closely related to the absorption of dietary lipids. First, these bioactive compounds must be released from the food matrix (i.e., the lipid phase surrounding the vitamins must be digested) [15,16]. Dietary lipids, like SO, are mainly composed of triacylglycerols (TGs), which are water-insoluble macromolecules that cannot be transferred from the intestinal lumen to the enterocytes. The ingested TGs must be emulsified and hydrolysed to monoacylglycerols (MGs) and free fatty acids (FFAs) to be absorbed in the GIT [17]. The pancreas is the main source of lipid-digesting enzymes, and the primary site of TGs hydrolysis is therefore the first part of the small intestine, the duodenum. However, the stomach also plays an important role in lipid digestion since its peristaltic movements induce the formation of O/W emulsions [18], and gastric lipase can hydrolyse part of the TGs ingested [19,20]. When lipids enter the duodenum, bile and pancreatic juice are released. Pancreatic lipases hydrolyse TGs to diacylglycerols as intermediates, and MGs and FFAs as final products. Bile salts increase the efficacy of lipolysis, increasing the surface area of oil–water interfaces at which water-soluble lipase is active [21]. The products generated from lipid digestion interact with bile salts, phospholipids, and other lipophilic compounds to form mixed micelles, which are transported across the mucus layer to the brush border membrane of the enterocytes where they are absorbed [22]. Lipophilic vitamins like α-toc must be incorporated in the hydrophobic interiors of those mixed micelles to be absorbed by the enterocytes, and secreted into the blood stream via the lymphatic system. Bioaccessibility of lipophilic vitamins like α-toc therefore depends on the total amount of mixed micelles and their nature [23]. Variations may also be due to the chemical nature of the vitamin, since tocopherol esters must be further hydrolysed by digestive enzymes before absorption [24]. To improve the bioavailability as well as the protection from oxidation of lipophilic compounds, different techniques can be employed. Lipophilic contents could be entrapped in the double layer of liposomes; this method has the advantage of being therapeutically efficient but it is also characterized by several drawbacks, such as low process replicability, low encapsulation efficiency, and large particles size distribution [25]. Encapsulation techniques based on drying processes are often employed and, among them, spray drying is one of the most widely used in the food industry due to its low-cost and flexibility [26]. Encapsulation by spray drying with β-glucans may increase the stability and availability of bioactive compounds; in fact, Ahmad et al. [27] discovered that the stability of saffron anthocyanins during passage through simulated GIT conditions was increased by using β-glucans extracted from barley. In addition, the bioavailability of anthocyanins in the intestinal section was increased. However, since the source, molecular weight, molecular structure, and types of bonding interactions have effects on the physicochemical and nutraceutical properties of β-glucans, further studies are required to better understand the behaviour of β-glucans from different sources, such as *P. ostreatus* mushroom.

Oxidative stability of oils has been defined as the resistance to oxidation not only during processing, but also during storage [28]. Resistance to oxidation can be expressed as the period of time required to reach the critical point of oxidation, whether it is a sensorial alteration or a rapid acceleration of the oxidative process. Oxidative stability is a significant indicator to determine shelf life and oil quality, since the low-molecular-weight off-flavour compounds produced through oxidation make oil undesirable to consumers or for industrial use as a food ingredient [29]. In order to implement lipophilic nutraceuticals enriched foods within the perspective of a value chain approach, long-storage stability is crucial; however, because of their high instability to oxidative deterioration, the storage of foods enriched with these compounds has been technically challenging [6,30].

In the first part of the study, the stability of α-toc in the GIT was investigated in vitro using the standardized INFOGEST method [31,32]. Additionally, the variations in size of the oil droplets throughout the different phases of the simulated GIT (oral, gastric and intestinal) were measured. The second objective of this work was to study the oxidative stability of SO, free or encapsulated in powder, with or without β-glucans rich extracts from *P. ostreatus*, correlated with α-toc degradation, in order to explore the potential protective effect exercised by the addition of a β-glucan-rich extracts on the oxidative status during storage. Our goal was to obtain a well-designed delivery system that can encapsulate bioactive lipophilic compounds in order to protect them from degradation during storage and digestion while ensuring satisfying bioavailability.

## 2. Materials and Methods

### 2.1. Materials

MD DE 12 (Glucidex^®^, Roquette, Lestrem, France) were used as the main wall material for the encapsulation process. One control emulsion (Ec_AG), was stabilized by adding AG (Instantgum AA, Nexira, Serqueux, France), while for the other control emulsion (Ec_Tween), Tween^®^ 20 (Sigma Aldrich, Saint-Quentin Fallavier, France) was used as surfactant [33]. Sample emulsions were stabilized with two β-glucan-rich extracts obtained from *P. ostreatus* (IoBoscovivo, Vergiate, VA, Italy) as described in a previous study [7] and labelled as (W) and (UW). Commercial SO containing 60% *w/w* polyunsaturated, 29% *w/w* monounsaturated and 11% *w/w* saturated fatty acids and 0.05% *w/w* α-toc (Cora, FR) was used as a model for lipophilic compounds’ encapsulation in powders. Free SO was also used for accelerated thermo-oxidation, together with the five powders Pc_Tween, Pc_AG, P10W, P10UW and P20UW, obtained after spray drying production in similar conditions as previously described [7,33]. The powders’ composition is described in Table 1.

For in vitro digestion, α-amylase (A1031, measured activity ≈ 1330 IU/mg) from human saliva, pepsin (P6887, measured activity ≈ 2820 U/mg) from porcine gastric mucosa, pancreatine (P1750, measured activity ≈ 3.416 U/mg) from porcine pancreas, bile porcine extract (B8631), Pepstatine-A (P5318), and Pefabloc (P76307) were obtained from Sigma-Aldrich (MO, USA). Standard α-toc (VWR International PBI, IT) was used for HPLC calibration. Different solvents were used to perform analysis: hexane (quality CHROMASOLV^®^) and isopropanol (quality CHROMASOLV Plus) were obtained from Sigma-Aldrich (FR). Tetrahydrofuran (THF), methanol (quality LC-MS), *n*-heptane and isooctane were purchased from Carlo Erba Reagents (Val-de-Reuil, France). All other chemicals used were purchased from Sigma Aldrich (Milan, Italy).

### 2.2. Methods

#### 2.2.1. In Vitro Digestion

Reconstituted emulsions (Ec_AG, E10W, E10UW and E20UW from Pc_AG, P10W, P10UW and P20UW, respectively) were prepared by mixing 5 g of each powder sample with 7.5 mL of distilled water (corresponding to the same concentrations as the emulsions before drying), stirred for 5 min at room temperature and immediately analysed for oil droplet size distribution (1 mL, Section 2.2.2) and in vitro digestion (5 mL). The in vitro digestibility of these reconstituted emulsions was then immediately assessed according to the guidelines of the INFOGEST network [31,32]. The digestion process was divided into three phases, called oral, gastric and intestinal phases. For each phase, the composition and pH of the simulated digestive fluids were replicated according to the INFOGEST recommendations and enzyme characterization assays were performed to determine enzyme activities [31,32]. The temperature was kept at 37 °C during the entire digestion process and preheated solutions were used during the procedure to avoid temperature variations.

For the oral phase, an amount of 9 g of reconstituted emulsion was mixed in a 50 mL conical tube with 7.2 mL of Simulated Salivary Fluid (SSF) stock solution, and pH was adjusted to 7.0 if needed (with a 1 M NaOH solution or a 1 M HCl solution, accordingly). Next, 45 μL of a 0.3 M CaCl_2_ solution, 0.5 mL of salivary amylase solution (75 IU/mL), and distilled water were added to reach a total volume of 18 mL. The tube was placed in a dry block incubator at 37 °C and 400 rpm for 2 min. At that point, 6 mL of the sample were withdrawn and immediately analysed (Section 2.2.2) or mixed with a hexane/iso-propanol solution (3:1, *v/v*) to inactivate the enzymes before further analysis (Section 2.2.4 and Section 2.2.6). Finally, the pH of the remaining oral phase mixture was lowered to 3 to inactivate the amylases.

For the gastric phase, an amount of 9.6 mL of Simulated Gastric Fluid (SGF) stock solution was added to the oral phase mixture, pH was checked, and adjusted if needed, before adding 6 μL of a 0.3 M CaCl_2_ solution, 0.5 mL of pepsin solution (2000 U/mL) and distilled water to reach a final volume of 24 mL. The mixture was incubated at 37 °C and 400 rpm for 2 h. At that point, 11 mL of sample were withdrawn and mixed with 110 μL of Pepstatin-A to inhibit the pepsins before further analysis. Finally, the pH of the remaining gastric phase mixture was increased to 7 to inactivate the enzymatic activity.

For the intestinal phase, an amount of 7.4 mL of Simulated Intestinal Fluid (SIF) stock solution was added to the gastric phase mixture, pH was checked, and adjusted if needed, before adding 26 μL of a 0.3 M CaCl_2_ solution, 1 mL of 10 mM bile extract, 3 mL of pancreatin solution (100 TAME U/mL) and distilled water to reach a final volume of 26 mL. The resulting mixture was incubated at 37 °C and 400 rpm for 2 h. At that point, 23 mL of sample were withdrawn and mixed with 230 μL of Pefabloc to inactivate the pancreatic enzymes before further analysis.

In vitro digestion of the reconstituted emulsions was repeated three times for each sample.

#### 2.2.2. Oil Droplet Size Distribution

The oil droplet size distribution of emulsions before and during in vitro digestion was measured by LASER light diffraction (Mastersizer 2000; Malvern, Orsay, France) in wet mode (Hydro 2000) after dispersion in distilled water. For the oil droplets, the refractive index value used was 1.475. The number weighted size distribution was used to study the particle size distribution of the emulsions, as well as the surface area moment mean D[3,2] and the volume moment mean D[4,3]. The D[3,2] is mostly sensitive to the presence of fine particles in the size distribution, while the D[4,3] is the most sensitive to the presence of large particles in the size distribution; D[3,2] was also studied in order to estimate the impact of the surface area of particles on oil oxidation.

#### 2.2.3. Accelerated Thermo-Oxidation

The spray dried powders were collected in polyethylene bags suitable for vacuuming, 0.20 mm thickness, and then stored under vacuum at −20 °C in the dark until use. In order to evaluate their oxidative stability and the protective effect exercised by β-glucan-rich extracts, an accelerated storage study was performed as follows: 30 g of each powder were equally distributed in three open glass Petri dishes (10 g powder/petri dish). The petri dishes were casually placed into a climatic test chamber (HC 0020, Vötsch Industrietechnik GmbH, Balingen-Frommern, Germany) at 60 °C and 50% relative humidity (RH) for 15 days. Samplings were done on triplicate at different times. When analyses of some samples needed to be delayed for practical reasons, samples were stored in sealed plastic bags under vacuum at −20 °C in the dark for few days. In a previous study, it was checked that no evolution regarding oxidation happened during frozen storage for powders [1]. In order to evaluate the oxidative stability of free oil, SO was equally distributed in three open glass Petri dishes, forming a thin layer and stored at the same conditions as the powders.

According to Lee et al. [3], activation energies of 79.496 kJ/mol and 83.680 kJ/mol were assumed for peroxide formation and degradation of tocopherols in SO, respectively. In order to predict storage at room temperature (20 °C) based on the data obtained at 60 °C, the Arrhenius law was applied, in order to find the acceleration factor of peroxide formation in SO (1):

(1) $$\frac{k60{\;}^{{}^{\circ}}C}{k20{\;}^{{}^{\circ}}C}=50$$

and degradation of tocopherols in SO (2):

(2) $$\frac{k60{\;}^{{}^{\circ}}C}{k20{\;}^{{}^{\circ}}C}=62$$

Hence, one day storage at 60 °C can then be considered equivalent to 50 days and 62 days for peroxide formation and degradation of tocopherols in SO, respectively, at 20 °C. Thus, in the present study a period of 15 days at 60 °C was chosen for the accelerated storage tests, highlighting the quality deterioration of SO, and simulating a shelf life of 2 years and 26 days for peroxide formation in SO and of 2 years and 200 days for degradation of tocopherols in SO at 20 °C.

#### 2.2.4. Oil Phase Extraction

The oil phase extraction from powders at different days of storage was performed according to the method described by Gallotti et al. [7]. To reconstitute the emulsions, 2 mL of purified water at 30 °C were added to 0.5 g of powder in a conical tube and agitated. Then, 50 mL of hexane/iso-propanol (3:1, *v/v*) were added to the reconstituted emulsion. The mixture was placed in an ultrasonic bath for 15 min and centrifuged at 1000× *g* for another 15 min at 20 °C. After centrifugation, around 47 mL of organic phase were recovered, where 40 mL was used for α-toc analysis and 1 mL was used to measure CD. The oil phase extraction from oral, gastric and intestinal digestion of powders was performed according to the same method with a few modifications. In brief, 2.5 mL, 5 mL and 10 mL of oral, gastric and intestinal phase, respectively, were withdrawn from the vials used for in vitro digestion (Section 2.2.1). These quantities were selected taking into account the dilution factor of every digestion phase. Then, 50 mL of hexane/iso-propanol (3:1, *v/v*) were added to the samples. The mixture was placed in an ultrasonic bath for 15 min and then centrifuged at 1000× *g* for 15 min at 20 °C. After centrifugation, around 47 mL of organic phase were recovered, where 40 mL were used for α-toc analysis.

#### 2.2.5. Measurement of CD

For measurement CD in free SO, 1 drop (e.g., 15–20 mg) was weighed in a 10 mL volumetric flask adding isooctane to get to volume; then, the vial was vortexed for 60 s. The sample absorbance was measured using a UV–visible spectrophotometer (UVIKON 941 Plus, Serlabo Technologies, Entraigues-sur-la-Sorgue, France) and the specific absorbance (SA) was calculated according to the following Equation (3):

(3) $$\mathrm{SA}=\frac{\mathrm{Abs}234\;\mathrm{nm}}{\mathrm{Wg}}$$

where Abs234 nm is the absorbance of the sample measured at 234 nm and Wg is the SO mass (g) in 100 mL of the solvent solution analysed.

For oil encapsulated by spray drying, measurements were made directly on the organic phase, which contained the extracted SO. For this purpose, 1 mL of the organic phase, extracted as described above (Section 2.2.4), was diluted 1:6 by adding 5 mL of hexane/iso-propanol (3:1, *v/v*), and was vortexed for 30 s. The SA was calculated according to the following Equation (4):

(4) $$\mathrm{SA}=6*\frac{\mathrm{Abs}234\;\mathrm{nm}}{\frac{g\;\mathrm{Calculated}\;\mathrm{Oil}}{47\;\mathrm{mL}}*100\;\mathrm{mL}}$$

where Abs234 nm is the absorbance of the sample measured at 234 nm; g Calculated Oil is the calculated mass of oil contained in 0.5 g of powder and recovered in 47 mL of organic phase.

Results were expressed according to the following Equation (5):

(5) ΔSA=SA(t)−SA(0)

where SA(*t*) is the SA at a specific storage day (day *t*) and SA(0) is the SA at the beginning of storage (day 0).

#### 2.2.6. Measurement of α-Toc Concentration

The chromatographic determination of α-toc was performed on a HPLC system as previously described by Gallotti et al. [7]. To measure the α-toc in the oil phase of powders, a precise volume (40 mL) of the organic phase, obtained as described above (Section 2.2.4), was evaporated in a flask by a rotary rotavapor (Heidolph Laborota 4000 efficient, WB eco, Schwabach, Germany). Then, 5 mL of methanol were added and the flask was placed in an ultrasonic bath for 5 min. The solution was filtered through a nylon syringe membrane with 0.45 μm pore size and 10 μL of the sample were injected and analysed using a model Shimadzu LC-20 AD pump coupled to a model Shimadzu SPD-M20A photodiode array detector and an RF-20 AXS operated by Labsolution Software Shimadzu, JP. A 5 μm Sunfire C18 column (250 × 4.6 mm; Waters, Milan, Italy) equipped with a C18 precolumn (Waters, Milan, Italy) was used for the separation, performed by isocratic elution thermostated at 28 °C, at a flow rate of 1 mL min-1 with a runtime of 35 min, using methanol/water (95:5, *v/v*) as mobile phase. Fluorimetric detection was made at an excitation wavelength of 290 nm and an emission wavelength of 330 nm. To measure α-toc content of free SO, one drop was weighed in a 10 mL volumetric flask and methanol was added to complete the volume. The flask was then vortexed for 60 s and placed in an ultrasonic bath for 5 min. The solution was filtered through a nylon syringe membrane with 0.45 μm pore size and 10 μL of the sample was injected and analysed as described for the powders. To measure the α-toc in the oil phase of digested powders, a precise volume (40 mL) of the organic phase was evaporated in a rotary rotavapor RE 120 (Buchi, Flawil, Switzerland). Then, 5mL of *n*-heptane was added to the extracted oil and the flask was vortexed for 60 s and placed in an ultrasonic bath for 30 s. The solution was filtered through a nylon syringe membrane with 0.45 μm pore size and put into vials. Then, 20 μL of the sample was injected and analysed with an HPLC system (Waters^®^, Milford, MA, USA) equipped with a pump (Waters^®^ 2695) coupled with a UV–visible diode array detector (DAD) at 298 nm (Waters^®^ 996). The stationary phase consisted in a bonded silica column 100 Diol (Lichrosphere, length 250 mm, internal diameter 4 mm, particle size 5 μm), thermostated at 25 °C and equipped with a precolumn (13 mm) with similar characteristics. The mobile phase was a mixture of *n*-heptane/tetrahydrofuran (96.15/3.85, *v/v*) eluted isocratically at a flow rate of 1 mL/min with a runtime of 12 min. A calibration curve was built with a purified standard was used for the identification and quantification of α-toc.

The degradation of α-toc was expressed according to the following Equation (6):

(6) $$\alpha -\mathrm{toc}\;\mathrm{degradation}\;\left(\%\right)=100*\frac{C0-Ct}{C0}$$

where C0 is the concentration of the component at the beginning of storage (day 0) and C*t* is the concentration measured at a specific storage day (day *t*).

#### 2.2.7. Statistical Analysis of Data

Experimental data were investigated using one-way ANOVA with the least significant difference (LSD) as a multiple range test, and by linear regression analysis using Statgraphics 5.1 (STCC Inc., Rockville, MD, USA). These results are reported as the average of triplicate values ± standard deviation (SD).

## 3. Results

### 3.1. Effect of In Vitro Digestion on Emulsion Structure

**Figure 1:**
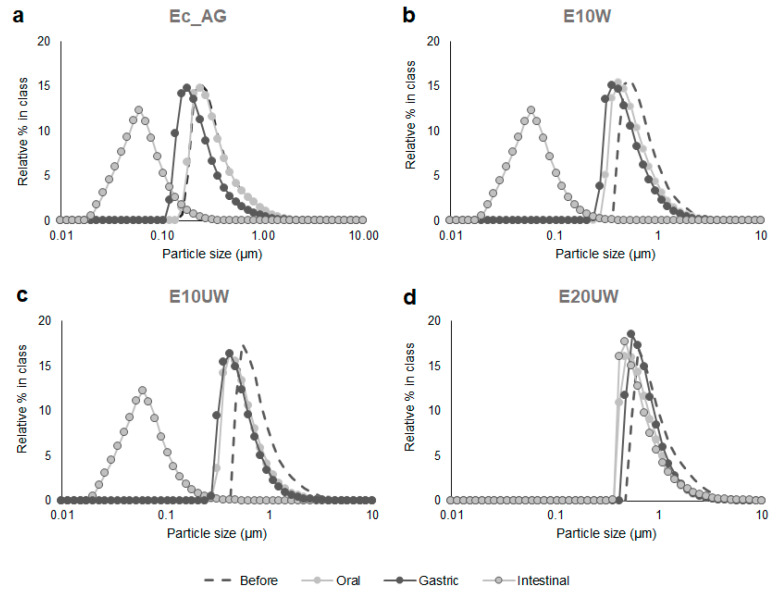
Effect of in vitro digestion on the number weighted particle size distributions of SO reconstituted emulsions stabilized with AG (**a**), β-glucan-rich extracts washed (**b**), and unwashed (**c**,**d**). Sample codes are explained in Section 2.2.1. Results presented are representative data for three separate experiments.

Particle size distribution of the four reconstituted emulsions was measured before in vitro digestion and after each stage of the INFOGEST protocol. The emulsions had a different structure prior to digestion. Ec_AG had the smallest oil droplets D[3,2] = 0.81 ± 0.01 µm and E20UW the largest D[3,2] = 2.64 ± 0. µm (Table 2). This was expected since E20UW had a higher oil content than the other samples, which is known to influence emulsion size [35]. Oil droplets in the three emulsions stabilized with the β-glucans-rich extracts from *P. ostreatus* were larger than the oil droplets in the emulsion stabilized with AG, as described in a previous study [7]. After the simulated oral phase, no significant changes were observed in the particle size distribution of Ec_AG (Table 2), meaning that the salivary amylase did not affect the structure of the emulsion. Particle size distributions were slightly shifted to lower values for E10W and E10UW (Figure 1b,c), and an increase in D[4,3] was measured for E20UW (Table 2). These results suggest that these emulsions were less stable than Ec AG after exposure to the oral phase conditions. This may be related to the content in MD of the original powders (Table 1). Salivary amylase in the simulated oral fluid may have partially hydrolysed MD used as wall material, leading to the disruption of some oil droplets (E10W and E10UW) or the coalescence of the largest less stable oil droplets (E20UW).

After the simulated gastric phase, the particle size distribution of Ec_AG was slightly shifted to lower values (Figure 1a), but no significant changes were observed in D[3,2] and D[4,3] (Table 2). These results agree with previous studies on emulsions stabilized by AG [36,37]. AG is an amphiphilic polysaccharide-based emulsifier, which attaches on the surface of the oil droplets and forms a thick interfacial layer resisting aggregation over a wide range of conditions, such as highly acidic conditions, through strong steric repulsions [37,38,39]. Similar results were observed for E10W after exposure to the gastric phase conditions (Figure 1b, Table 2). Consequently, for the control sample (Ec_AG) and the sample containing the W extract (E10W), the structure of the emulsion that reached the intestinal phase was similar to the structure of emulsion before in vitro digestion. In contrast, an increase in D[4,3] was measured for E10UW after the gastric phase (Table 2), meaning that the amount of large oil droplets in the emulsion increased. This partial coalescence is probably due to the protonation of carboxyl groups on the polar regions of the emulsifier (i.e., proteins associated to β-glucans in the mushroom extracts) under highly acidic conditions, leading to a decrease in electrostatic repulsions between the oil droplets. However, the majority of the oil droplets were still resistant to aggregation (Figure 1c), suggesting that they were mostly protected by steric repulsions. No further changes were observed for E20UW after exposure to the gastric phase conditions (Table 2).

Overall, both W and UW extracts from *P. ostreatus* were able to stabilize the emulsions during the first phases of the digestion in vitro. More coalescence was observed in sample E10UW than in sample E10W, which may be related to the different molecular weights of the β-glucans in the extracts [7].

After the simulated intestinal phase, it should be noted that a significant increase in D[4,3] was measured for all samples (Table 2). This was attributed to the presence of large insoluble bile particles and pancreatin filaments in the simulated intestinal fluid (D[3,2] = 82.25 ± 11.45 µm and D[4,3] = 461.61 ± 10.58 µm).

At the end of the in vitro digestion, a significant decrease in particle size was observed for Ec_AG, E10W and E10UW (Figure 1a,b,c), which can be attributed to the hydrolysis of the oil droplets by pancreatic enzymes and the formation of mixed micelles by bile salts and lipid digestion products. The mixed micelles produced from the in vitro digestion of Ec_AG, E10W, and E10UW were similar in size (mode ≈ 60 nm). Tan et al. [37] also observed the formation of mixed micelles after in vitro digestion of β-carotene loaded corn oil O/W emulsions stabilized with AG. On the other hand, the D[3,2] value measured for E10UW at this stage was higher than for E10W. Therefore, it appears that E10UW was less digested by the pancreatic lipases than E10W. This may be related to irreversible coalescence in the gastric phase, since the size of the oil droplets is known to influence the kinetics of lipolysis. Larger droplets have a smaller surface area available for interaction with lipase molecules, resulting in a decrease in the rate of lipolysis [40,41].

No significant changes were observed in the particle size distribution of E20UW at the end of the in vitro digestion (Figure 1d). However, an increase in D[3,2] was noted (Table 2), probably due to partial droplet coalescence. These results suggest a reduction in the physical stability of E20UW during this last phase of digestion. Bile salts and phospholipids present in the simulated intestinal fluid may have displaced some of the β-glucans from the oil droplet surfaces, leading to droplet coalescence and a decrease in the rate of lipolysis. E20UW was also probably the less stable emulsion due to its higher oil content. Indeed, when increasing the oil content, the wall material content decreases (for the same total solids content), which may lead to faster droplets coalescence [42]. Similar results were reported by Jeanes et al. [16] for O/W emulsions made with corn oil, α-toc acetate and quillaja saponins. However, they also pointed out that the overall influence of lipid digestion on oil droplets is complicated and may result in an increase or a reduction in their size, since the digestion products of lipolysis may accumulate at the oil–water interface or move into the surrounding aqueous phase, depending on their molecular weight and the concentrations of bile salts and phospholipids [22,23].

### 3.2. Effect of In Vitro Digestion on α-Toc Stability

**Figure 2:**
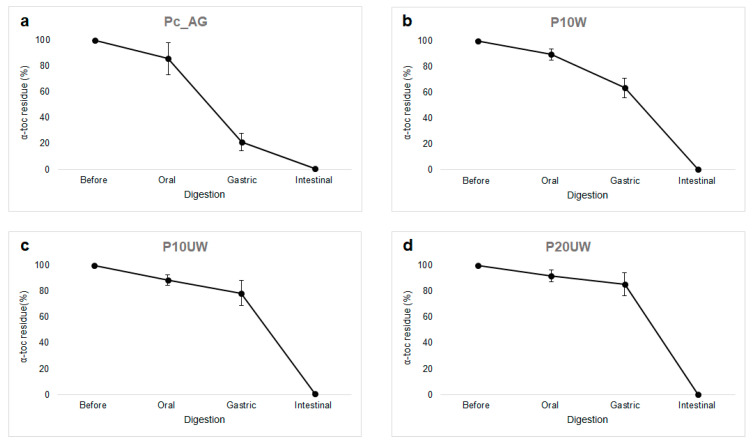
Effect of in vitro digestion on the vitamin E degradation in SO reconstituted emulsions stabilized with AG (**a**), β-glucan-rich extracts washed (**b**), and unwashed (**c**,**d**). Sample codes are explained in Section 2.2.1. Results presented are representative data for three separate experiments.

Lipophilic bioactive compounds, such as α-toc, are usually situated inside of the lipid droplets and, in order to be released, the surrounding TGs have to be digested. After being released from the droplets, they have to be incorporated into the hydrophobic areas within the mixed micelles; if not, they will precipitate or form a separate layer [43]. To assess the suitability of Ec_AG, E10W, E10UW and E20UW as emulsion-based delivery systems for lipophilic compounds, presence of α-toc in the oil phase were examined after in vitro oral, gastric and intestinal digestion (Figure 2).

During mastication, oxygen can be incorporated and triggers oxidative degradation of lipophilic compounds within the food matrix. Hence, the possible influence of the oxygen on the incidence of lipid oxidation cannot be excluded, since it is a mayor limit for in vitro and ex vivo models [13]. However, in all samples there was no significant decrease in α-toc.

While no significant changes were observed in the particle size distribution of Ec_AG, α-toc concentration in this sample significantly (*p* < 0.05) decreased compared to the samples with β-glucans. The α-toc residue was 21% in Ec_AG, 63% in E10W, 79% E10UW and 86% in E20UW. Such difference could be explained by the fact that β-glucans acted as an antioxidant and protected α-toc against the low pH of the simulated gastric juice, which is a well-known pro-oxidant condition of the GIT [13].

Since most nutrients and vitamins are absorbed at the intestinal level for additional utilization in the body, α-toc should be preserved in encapsulated particles and should not be released in the stomach [44]. At the beginning of the simulated intestinal phase, the residual amount of vitamin E in the digesta of E10W, E10UW and E20UW was not significantly different from the amount recovered in the oil phase before digestion. Therefore, these samples had a better vitamin E stability during the first two phases of in vitro digestion compared to the control sample Ec_AG. However, after 2 h of intestinal digestion, no α-toc was found in any of the four samples (Figure 2). It is possible that the presence of reactive species like metallic ions activated some oxidative degradation phenomena; moreover, the products of the enzymatic hydrolysis of food lipids are prone to oxidative degradation. Since large oil droplets have a smaller surface area available for lipolysis and oxidation, different results were expected in samples containing UW extracts. However, no α-toc was found in the digesta of these samples either. Thus, no correlation between changes in size of the oil droplets and α-toc stability was observed.

However, it is not simple to explain this phenomenon, as physical stability and bioactive bioaccessibility are elaborated processes influenced by several factors. It is well known that polysaccharides can interact with lipase, bile acids, calcium ions and other various digestive components [45]. Therefore, Lv et al. [43] made assumptions that the AG interacts with bile salts and/or free fatty acids, thus decreasing the incorporation of α-toc into the mixed micelles or causing the latter to precipitate and thereby not be detectable after the oil phase extraction. This may have occurred here with *P. ostreatus* β-glucans, as an increase in turbidity was observed in the samples after the intestinal stage. Clearly, further in vivo studies are required to better understand the results obtained using this in vitro model, especially regarding the intestinal step.

### 3.3. Oxidative Stability during Storage

**Figure 3:**
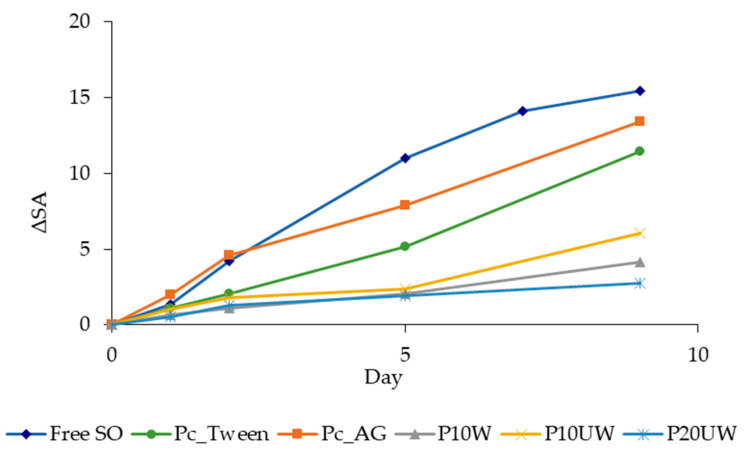
CD (expressed as ΔSA = SA(t) − SA(0) value) of Free SO and of spray dried emulsions, determined at different storage times up to 9 days. Sample codes are explained in Table 1.

Oxidative stability of oils has been defined as the resistance to oxidation during processing and storage [28]. For comparison purposes, free SO, the control powders Pc_AG and Pc_Tween, and powders made with *P. ostreatus* β-glucans, were stored at 60 °C and 50% RH in air in order to study the CD and antioxidant (α-toc) content under thermo-oxidation conditions. The CD are formed by the rearrangement of the hydroperoxide double bonds during oxidation, thus representing the primary degradation products of oil [46]. The CD content of free SO and of the different powders is reported in Figure 3. Although spray drying encapsulation was expected to protect PUFAs from oxidation during accelerated storage, evolutions of CD content for the control powders Pc_Tween and Pc_AG showed a fast increase during the first days of storage (Figure 3), up to 11.5 and 13.4, respectively, in 9 days (Table 3). The CD content in the free oil reached 15.5 in 9 days and then the oil appeared as a resin, indicating that polymerization occurred. However, spray drying encapsulation prevented the polymerization of encapsulated oil. The presence of β-glucan-rich extracts gave higher protection compared to the control powders, made with emulsifier with no recognized antioxidant activity (Figure 3). The CD of P10W and P20UW increased similarly, both reaching 2 after 5 days, and 4.2 and 2.8 after 9 days, respectively. P10UW also reached a CD value around 2 after 5 days, then it slightly increased up to 6 at day 9 of storage, which is about half the CD values of the control powders. The storage of free oil was stopped at 9 days because further analyses were not possible since the oil could not be dissolved into isooctane. Conversely, the storage study continued for an additional 6 days with the powders only (Table 3). Along the 15 days of storage, the amount of CD for all samples increased; however, β-glucans clearly acted as an antioxidant. The maximum CD levels for P10W and P10UW reached at 15 days were 9.6 and 11.3, respectively, showing no significant differences, while P20UW reached the maximum of 6.6 (Table 3). Consequently, for P10W, P10UW and P20UW, the increase in CD content during the first days of aging was slower, suggesting that β-glucan-rich extract played a role in the prevention of the formation of CD from peroxyl radicals, thus slowing down the propagation stage of oxidation. Regarding the control powders, Pc_Tween reached the maximum of CD content after about 12 days of aging, followed by a stabilization (CD around 15), while Pc_AG had a notable decrease immediately after reaching the maximum CD level (Table 3). In SO, when the concentration of hydroperoxides is significant, secondary oxidation products are formed [28]. Thus, in Pc_AG, the CD level fell because all the hydroperoxides were oxidized to aldehydes and ketones, while this did not happen in the other powders during 15 days of storage.

To further study the behaviour of SO concerning oxidation, the time-course of α-toc consumption in free and encapsulated SO was analysed (Table 3). α-toc was consumed progressively with different rates depending on the sample. After 9 days of aging, the consumption of α-toc in free SO was completed (99%), while spray drying encapsulation with Tween 20 delayed the degradation (67%) and the presence of *P. ostreatus* β-glucans slowed it down by between 18 and 25%. In the other control powder, made with AG, the consumption of α-toc was even faster than in free SO, with higher percentages of degradation since the first days of storage. In a previous study on β-carotene stability in O/W emulsions with different droplet sizes, it was demonstrated that the degree of degradation of this oxygen-sensitive target increases with a decrease in mean particle diameter. This effect was attributed to the increased surface area of the smallest droplets with respect to the larger droplets and the bulk lipid phase [47]. Hence, the fast oil CD formation in Pc_AG, similar to the free oil, and the even faster decrease in α-toc content with respect to the free oil, can be explained by the fact that Pc_AG had the smallest D[3,2] (9.39 ± 0.36 µm) and hence it was more accessible to oxygen. Conversely, Pc_Tween had higher D[3,2] (16.10 ± 0.97 µm) and provided better oil protection than Pc_AG. Interestingly, P10W, P10UW and P20UW with D[3,2] of 11.09 ± 4.69, 17.50 ± 1.28 and 14.63 ± 5.71 µm were able to retain a significant percentages of α-toc through storage under accelerated conditions. After 5 days of storage, the retention of α-toc in P10W, P10UW and P20UW was 82%. As explained in the Materials and Methods section, the predicted variation of α-toc is 62 times slower at 20 °C than under the accelerated conditions and hence 82% retention can be expected after 310 days at 20 °C, i.e., approximately one year. At the end of the storage, α-toc was completely consumed in the control powders and in free SO, while in both P10W, P10UW and P20UW the percentage of α-toc residue was about 70%. The powders made with β-glucan-rich extracts showed the lowest α-toc degradation values during storage, with no significant differences between them, confirming that the presence of an emulsifying agent with inherent antioxidant activity can help in the protection of bioactive compounds susceptible to oxidation.

## 4. Conclusions

The purpose of this study was to determine the influence of different emulsifying agents on the stability of encapsulated SO, rich in PUFAs and vitamin E, using in vitro digestion and an accelerated storage. The powders obtained by adding the β-glucan-rich extracts appeared to have better oxidative stability, with low α-toc degradation and production of CD, whereas those stabilized by the commonly used emulsifier (i.e., AG and Tween 20) were prone to oxidation. The β-glucan-emulsions had similar vitamin E degradation profiles during in vitro digestion, whereas degradation was faster in the AG-emulsion in the gastric phase. This phenomenon was linked to the antioxidant properties of β-glucans, thereby inhibiting the oxidation phenomena until the beginning of the intestinal phase. This study has proved for the first time the potential antioxidant activity of β-glucan used as emulsifying agent under in vitro gastrointestinal digestion and accelerated aging conditions. However, no α-toc was found at the end of the intestinal phase in any of the digested samples, probably because of some oxidative degradation phenomena. The structure of the emulsions during in vitro digestion depended on the emulsifier used and the oil content. Emulsions were better protected from aggregation during the simulated oral and gastric phases by AG, but the mushroom-derived emulsifiers were also able to fairly stabilize the oil droplets. Both types of emulsifiers led to the formation of mixed micelles during the simulated intestinal phase, but when the oil content was increased the stability of the emulsion decreased, and large oil droplets were observed instead of mixed micelles. Further studies are needed to better understand the fate of vitamin E encapsulated in O/W emulsions. In particular, the kinetics of α-toc degradation during the intestinal phase and the presence of oxidation products during digestion should be studied in order to design emulsion-based delivery systems robust enough to last through food production, storage, and digestion; moreover, in vivo animal feeding studies are required to confirm the results of this in vitro study.

## Figures and Tables

**Table 1:** Composition of the powders used for the study and β-glucans and proteins content of dry matter in the extract.

Powder Code	Powder Composition					Extract d.m. Composition	
	Sunflower Oil (SO) (%)	Tween^®^ 20 (%)	AG (%)	MD (%)	Extract d.m. (%)	β-Glucans (%)	Proteins (%)
Pc_Tween	10	0.3	---	89.7	---	---	---
Pc_AG	10	---	36	54	---	---	---
P10W	10	---	---	89	1	0.21	0.21
P10UW	10	---	---	89	1	0.22	0.14
P20UW	20	---	---	78	2	0.43	0.29

Extract d.m. is the dry matter of the W and UW hot water extracts from *P. ostreatus* obtained as previously described [34].

**Table 2:** Oil droplet size distribution statistics in reconstituted emulsions before in vitro digestion and after the oral, gastric and intestinal phase of the INFOGEST protocol.

Emulsion Reconstituted	Digestion Stage	Particle Size					
		D (3,2)			D (4,3)		
**Ec_AG**	Before	0.81 ± 0.01	A	a	1.15 ± 0.01	A	a
	Oral	0.75 ± 0.05	A	a	1.13 ± 0.03	A	a
	Gastric	0.77 ± 0.06	A	a	1.13 ± 0.03	A	a
	Intestinal	0.68 ± 0.15	A	a	299.15 ± 100.12	B	a
**E10W**	Before	1.76 ± 0.01	A	b	2.58 ± 0.36	A	a,b
	Oral	1.67 ± 0.04	A	b	3.46 ± 0.43	A	a
	Gastric	1.53 ± 0.24	A	b	4.19 ± 1.58	A	a,b
	Intestinal	0.80 ± 0.10	B	a	305.13 ± 83.79	B	a
**E10UW**	Before	2.23 ± 0.02	A	c	4.16 ± 0.90	A	b,c
	Oral	1.88 ± 0.31	A	b	5.34 ± 3.60	A	a
	Gastric	2.01 ± 0.33	A	b	17.28 ± 1.46	B	c
	Intestinal	1.83 ± 0.56	A	a	407.13 ± 37.51	C	a
**E20UW**	Before	2.64 ± 0.05	A	d	4.37 ± 0.38	A	c
	Oral	2.45 ± 0.22	A	c	16.81 ± 6.63	B	b
	Gastric	2.68 ± 1.10	A	c	9.53 ± 3.08	B	b
	Intestinal	8.39 ± 3.05	B	b	233.05 ± 27.77	C	a

In the same column, significant differences (*p* < 0.05) are indicated by capital letters (A, B, C) when comparing different digestion stages, and by lower-case letters (a, b, c) when comparing different emulsions. Sample codes are explained in Section 2.2.1.

**Table 3:** CD content and degradation of α-toc in free SO and in powders, determined at different storage times of up to 15 days.

	Free SO			Pc_Tween			Pc_AG			P10W			P10UW			P20UW		
**Day**	**CD (ΔSA)**																	
**0**	0.0 ± 0.0	A	a	0.0 ± 0.0	A	a	0.0 ± 0.0	B,C	a	0.0 ± 0.0	A	a	0.0 ± 0.0	A	a	0.0 ± 0.0	A	a
**1**	1.4 ± 0.2	A	a	1.1 ± 0.1	A,B	a	2.0 ± 0.9	B,C	a	0.6 ± 0.6	A	a	1.0 ± 0.7	A,B	a	0.6 ± 0.4	A	a
**2**	4.2 ± 0.6	B	b,c	2.1 ± 0.2	B	a,b	4.6 ± 1.5	C	c	1.2 ± 0.5	A	a	1.8 ± 0.7	A,B	a,b	1.4 ± 0.7	A,B	a
**5**	11.0 ± 1.2	C	c	5.4 ± 0.4	C	a,b	7.9 ± 1.5	C,D	b,c	2.1 ± 0.5	A	a	2.4 ± 0.3	B	a	2.0 ± 0.2	A,B	a
**9**	15.5 ± 0.5	D	c	11.5 ± 0.3	D	a,b,c	13.4 ± 5.9	D	b,c	4.2 ± 1.1	B	a	6.1 ± 0.9	C	a,b	2.8 ± 0.2	A,B	a
**12**	n.d.			15.1 ± 0.6	E	d	−5.7 ± 0.4	A	a	7.0 ± 0.2	C	c	7.0 ± 0.8	C	c	4.0 ± 0.4	B,C	b
**15**	n.d.			15.4 ± 0.8	E	d	−3.3 ± 1.3	A	a	9.6 ± 0.1	D	c	11.3 ± 0.8	D	c	6.6 ± 0.1	C	b
**Day**	**Degradation of α-Toc (%)**																	
**0**	0 ± 0	A	a	0 ± 0	A	a	0 ± 0	A	a	0 ± 0	A	a	0 ± 0	A	a	0 ± 0	A	a
**1**	8 ± 15	A	a,b	1 ± 8	A	a	27 ± 6	B	b	3 ± 1	A	a	10 ± 5	A,B	a,b	7 ± 4	A	a,b
**2**	12 ± 11	A	a	22 ± 9	B	a	64 ± 3	C	b	8 ± 4	A	a	12 ± 4	A,B	a	16 ± 5	A	a
**5**	42 ± 11	B	b	50 ± 2	C	b	87 ± 2	D	c	17 ± 5	B	a	18 ± 3	B,C	a	17 ± 7	A	a
**9**	99 ± 0	C	c	67 ± 5	C	b	95 ± 1	D	c	18 ± 3	B	a	20 ± 5	B,C	a	25 ± 6	A	a
**12**	n.d.			95 ± 1	D	b	97 ± 1	D	b	20 ± 2	B	a	28 ± 3	C,D	a	28 ± 3	A	a
**15**	n.d.			98 ± 1	D	b	97± 1	D	b	27 ± 4	B	a	37 ± 3	D	a	29 ± 7	A	a

CD and degradation of α-toc (expressed as 100 × (C0 − Ct)/C0) in Free SO and in spray dried emulsions, determined during storage. Sample codes are explained in Table 1. n.d. = not detectable. Different capital letters (A, B, C, D, E) in the same column were used to designate significant difference (LSD, *p* < 0.05) for different storage times (same sample), and different lower-case letters in the same row (a, b, c, d) were used to designate significant difference among different samples (same storage time).

## Abbreviations

PUFAs, polyunsaturated fatty acids; SO, sunflower oil; α-toc, α-tocopherol; O/W, oil-in-water; MD, maltodextrins; AG, acacia gum; GIT, gastrointestinal tract; TGs, triacylglycerols; MGs, monoacylglycerols; FFAs, free fatty acids; SSF, Simulated Salivary Fluid; SGF, Simulated Gastric Fluid; SIF, Simulated Intestinal Fluid; RH, relative humidity; CD, conjugated dienes; SA, specific absorbance; DAD, diode array detector; LSD, least significant difference; SD, standard deviation.
